# Extracellular vesicles: biogenesis mechanism and impacts on tumor immune microenvironment

**DOI:** 10.1186/s12929-025-01182-2

**Published:** 2025-09-04

**Authors:** Nai Yang Yeat, Ruey-Hwa Chen

**Affiliations:** 1 Institute of Biological Chemistry, Academia Sinica, Taipei, 115 Taiwan; 2 Chemical Biology and Molecular Biophysics Program, Taiwan International Graduate Program, Academia Sinica, Taipei, 115 Taiwan; 3 Institute of Biochemical Sciences, College of Life Science, National Taiwan University, Taipei, 106 Taiwan

**Keywords:** Extracellular vesicles, EV biogenesis, EV secretion, Tumor immune microenvironment, Cancer therapy

## Abstract

Extracellular vesicles (EVs) are heterogeneous populations of membrane-bound particles released from almost all cell types in an organism and play pivotal roles in cell–cell communication. EVs carry nucleic acids, proteins, metabolites and other bioactive substances, which are taken by the recipient cells to alter cell physiology and functions. The cargo landscapes of EVs are influenced by the cell contexts and the biogenesis mechanisms of EVs, in which certain molecules govern both biogenesis and cargo sorting. In this review, we discuss the biogenesis and secretion mechanisms of various types of EVs, including several atypical EVs. In addition, given that the tumor immune microenvironment (TIME) is intricately controlled by the communication between tumor cells and various immune cells, we summarize the latest update about how tumor-derived EVs influence the phenotypes of various immune cells in tumor microenvironment for tumor immune evasion, and, conversely, how EVs secreted from immune cells in TIME control the malignancies of tumor cells. In particular, we discuss the roles of several atypical EVs in regulating TIME. Lastly, we highlight the advantages of utilizing EVs as liquid biopsies for cancer diagnosis, the application and challenge of EVs in different anti-tumor therapies, and the recent clinical trials that exploit EVs as drug carriers. As the continuous advances in our understanding of the complex biogenesis mechanisms and the pleiotropic actions of EVs in TIME as well as the technology improvements in harnessing EVs’ clinical benefits, we can expect to further unlock the biomedical potential of EVs in cancer and other diseases.

## Background

Extracellular vesicles (EVs) are nano-sized and membrane-bound particles released from cells into the extracellular space. EVs carry proteins, RNAs, DNAs, lipids, metabolites and other biologically active substances. These EV cargos are delivered to the recipient cells, located either in the vicinity of EV secretion site or in the distant tissues, thereby impacting on the functions of recipient cells. Thus, EV has been increasingly recognized as a key mediator of cell–cell communication. This function is particularly important in the tumor microenvironment (TME) [1], where tumor and stromal cells co-evolve and mutually educate to promote tumor progression.

EVs comprise a heterogeneous array of vesicles that are generated by diverse mechanisms. Based on their origins in the cellular compartments, EVs are categorized into two major types, i.e. ectosomes and exosomes [2, 3]. Ectosomes are generated by the outward budding of plasma membrane and include various types of vesicles with a large range of sizes, such as microvesicles (also known as small ectosomes; 0.1–1 m diameter) and large oncosomes (> 1 m). Exosomes, however, are small EVs (50–180 nm) originated inside of the cell through a divergence of the endocytic pathway. That is, the multivesicular bodies (MVBs) (also known as multivesicular endosomes) escape the degradation via lysosomes and fuse with plasma membrane, resulting in the release of intraluminal vesicles (ILVs) into the extracellular space as exosomes. Recently, a number of additional EVs types have been reported, including apoptotic bodies [4, 5], secretory autophagosomes/amphisomes [6–8], and migrasomes [9, 10]… etc. Thus, it has become increasingly difficult to discriminate EVs based on their biogenesis mechanism or site of origin. Since current isolation strategies of EVs allow the discrimination according to the size and density, the latest classification guidelines recommend the description of purified EVs based on their size: i.e., small EVs (sEVs; < 200 nm) and large EVs (lEVs; > 200 nm) [11], unless the biogenesis mechanism is well defined. Since EVs derived from different origins and biogenesis mechanisms likely carry different cargo repertories and undergo different fates in the recipient cells, it would be important to discriminate them to understand their physiological and pathological impacts and to manipulate their production for therapeutic applications.

In this review, we discuss the current knowledge on EVs biogenesis and secretion mechanisms. In addition, given the pivotal roles of EVs in governing the intercellular communications between tumor and immune cells in the TME, we provide extensive evidence for the effects of EVs on controlling tumor immunity and the recent advances in EV-based strategies for cancer therapy.

## Main text

### Biogenesis of exosomes

#### ESCRT-mediated MVB and exosome biogenesis

In the endocytic pathway, the inward budding of endosome limiting membrane generates ILVs inside the endosome, which leads to the maturation of endosome into MVBs. Since ILVs represent the pre-secreted exosomes, their formation is a major mechanism of exosome biogenesis. The canonical ILV generation mechanism involves the endosomal sorting complex required for transport (ESCRT) complex, in which the four subcomplexes, ESCRT-0, ESCRT-I, ESCRT-II, and ESCRT-III, are sequentially recruited to endosome membrane [12–16]. The ESCRT-0, ESCRT-I, and ESCRT-II subcomplexes coordinate cargo sorting with membrane bending. Each subcomplex contains ubiquitin-binding proteins for recruiting ubiquitinated transmembrane proteins to the endosome membrane, such as epidermal growth factor receptor whose ubiquitination is stimulated by ligand binding. In particular, the multiple ubiquitin-binding domains within the ESCRT-0 enable a high avidity binding and the ability of ESCRT-0 component HRS to recruit both ubiquitinated cargos and clathrin facilitates the clustering of HRS and ubiquitinated cargos within a sub-domain of the endosome limiting membrane for cargo sorting [17, 18]. Cargos are thought to transfer from ESCRT-0 to ESCRT-I and ESCRT-II [19–21], which also promote the bending of endosome limiting membrane to form ILVs [22]. Subsequently, ESCRT-III is recruited to form filament, which facilitates membrane bending and fission for ILV generation [23, 24]. Finally, AAA ATPase VPS4 removes ESCRT-III from the endosome membrane to complete the membrane scission event [25].

In addition to the canonical ESCRT pathway, the ESCRT accessory protein ALIX governs an alternative pathway for ILV generation and exosome biogenesis. The key player of this pathway is the syndecan-syntenin-ALIX tripartite complex [26]. Syndecan is a transmembrane proteoglycan containing heparin sulphate in its extracellular domain. On the endosome membrane, syndecan recruits PDZ domain-containing protein syntenin, which in turn binds ALIX. Importantly, the exosome biogenesis activity of syndecan-syntenin-ALIX complex also depends on ESCRT-I, ESCRT-II, and ESCRT-III. Given the interaction of ALIX with ESCRT-I component TSG101 and ESCRT-III component CHMP4B [27], ALIX likely functions as an alternative platform to organize ESCRT on the endosome membrane for ILV formation. Additionally, ALIX, syntenin and syndecan are all involved in cargo sorting during ILV generation.

#### Other molecules in MVB and exosome biogenesis

Exosome biogenesis can also be induced by lipids. Ceramide and phosphatidic acid induce negative membrane curvature on the endosome membrane through their cone-shaped structure, which promotes endosome membrane invagination to form ILVs [28, 29]. In line with this finding, inhibition of ceramide production by inhibitor of the neutral sphingomyelinase 2 impairs the sorting of proteolipid protein (PLP) into ILVs and exosomes. In addition, PLP in endosome is localized to the flotillin 2-containing membrane domain (a marker for caveolae/lipid raft), but not in the HRS-containing domain. Accordingly, PLP endosomal sorting is independent of several ESCRT components [29]. These findings support that ceramide mediates an ESCRT-independent exosome biogenesis pathway. However, ESCRT system may act in concert with ceramide for ILV generation, with the former providing sorting ability and the latter enhancing membrane bending. Indeed, it has been shown that ESCRT-II associates with the cholesterol-rich membranes in the reconstituted system to promote the formation of liquid-ordered domains (L_0_ domains), which are enriched in ILVs [30]. Thus, ESCRT pathway may depend on L_0_ domain environment for ILV generation. In addition, ceramide-dependent and ALIX-dependent pathways can be exploited by the same cell to generate two populations of EVs. In polarized epithelial cells, EVs secreted from the basolateral membrane are derived from a ceramide-dependent mechanism, whereas those from the apical membrane are originated from an ALIX-dependent mechanism [31].

Tetraspanin-family proteins such as CD63, CD9, and CD81, are canonical exosome markers. These proteins are commonly used to identify exosomes due to their enrichment in vesicles derived from the endosomal pathway. As a result, they are typically present at lower levels in other types of extracellular vesicles. However, tetraspanins are also involved in cargo sorting into exosomes [32]. Structural analyses have revealed that their four conserved transmembrane helices form a cone-like structure with an intramembrane cholesterol-binding pocket, suggesting a potential role in promoting membrane bending [33]. However, the exact function of tetraspanins in EV biogenesis remains elusive and conflicting observations have been reported [34]. In addition, tetraspanins can also be sorted into EVs through an ALIX- and ESCRT-III-dependent mechanism [35], suggesting a hierarchy of the endosome sorting mechanism.

In certain cancer cells, inhibition of mTOR/AKT pathway or deprivation of glutamine leads to the formation of a unique class of exosomes [36]. This type of exosomes is derived from Rab11-postivie recycling endosomes rather than the Rab5- or Rab7-positive endosomes, and therefore devoid canonical exosome markers. Formation of this type of exosomes involve ESCRT-III accessory proteins CHMP1, CHMP5, and IST1 [34].

### Biogenesis of ectosomes

#### Formation of small ectosomes (microvesicles)

The outward budding of plasma membrane is topologically similar to the inward budding of endosome membrane. Thus, it is not surprising that many molecules involving in exosome formation also play important roles in small ectosome formation, such as ESCRT, tetraspanins, and ceramide [37–39]. In the ESCRT-dependent ectosome biogenesis pathway, the ESCRT-I component TSG101 is recruited to HRS-like molecule arrestin domain-containing protein 1 (ARRDC-1) and cargo sorting is also mediated by ubiquitination [37, 39]. Furthermore, ESCRT-III and VPS4 are involved in membrane scission to release vesicles. Microvesicles generated by this mechanism is called ARRDC-1-mediated microvesicles (ARRMs). Similar to exosome formation, tetraspanins such as CD9 and CD81 are involved in bending of plasma membrane and sorting of cargos to microvesicles [38]. Because of the involvement of certain common molecules, it is difficult to define the origin of EVs simply by ablating one or two molecules.

In addition to the outward budding of plasma membrane, small ectosomes can also be generated by shedding of the membrane protrusions, such as microvilli and filopodia [34, 40]. This can occur in damaged sites of plasma membrane and undamaged cells. In addition to ESCRT components and cholesterol, the actin cytoskeleton remodeling proteins, such as the Rho/ROCK/myosin axis, and the extracellular and pericellular matrix components such as hyaluronan and glycocalyx, impact on the formation of EVs from the membrane protrusion [41–44]. In addition, proteins with the inverse BAR (I-BAR) domain, such as MIM, are enriched in the protrusion tips to induce membrane remodeling, thereby contributing to EVs generation [45].

#### Formation of large ectosomes

Current knowledge reveals that the generation of large vesicles from the plasma membrane involves the alteration of membrane compositions at the site of vesicle formation [34]. For instance, tumor-derived large ectosomes contain phosphatidylserine [46], which is likely resulted from the recruitment and activation of calcium-dependent lipid scramblase and floppase to disrupt the plasma membrane lipid asymmetry, leading to the externalization of phosphatidylserine [47, 48]. Similarly, externalization of phosphatidylethanolamine by inhibiting its flippase TAT-5 promotes large ectosome formation in *C. elegan*s embryos.

### Biogenesis of other types of EVs

#### Apoptotic bodies

Formation of apoptotic bodies is a character of cell apoptosis and is critical for apoptotic clearance and for communication between apoptotic cells and surrounding cells. Apoptotic bodies are large EVs and their formation involves three steps: plasma membrane blebbing, plasma membrane protrusion (called apoptopodia), and apoptotic body fragmentation [49]. Although the detailed mechanism is not fully understood, caspase 3 substrates such as ROCK, LIMK, and pannexin 1 are involved in the first and/or second steps of apoptotic body formation [50–53], whereas plexin B2 is responsible for apoptopodia beading [4], a phenomenon related to apoptotic body fragmentation.

#### Secretory autophagosomes/amphisomes

Secretory autophagy is defined as the requirement of autophagic machinery for the biogenesis of EVs and has been discovered in yeast and mammal [7]. In mammal, secretory autophagy is responsible for the secretion of cargos without leader peptides, such as cytokine IL-1β, aggregation-prone protein α-synuclein, and autophagic receptors p62 and NBR1 [8, 54, 55]. Importantly, autophagic proteins involving in different steps of autophagosome formation participate in the secretory autophagy, including phagophore formation (FIP200, ATG14), LC3-conjugation (ATG7, ATG12), and autophagosome sealing (ATG2A/B) [8]. Thus, it is believed that secretory autophagy is originated by fusing autophagosome-like vesicles with plasma membrane. Several lines of evidence support this notion. First, autophagosome can fuse with MVBs to generate a hybrid organelle called amphisome [56], which likely direct autophagosome to undergo both degradative and secretory fates as MVBs. Second, unlike general autophagy, secretory autophagy requires Rab8a and Rab27a, which mediate the transport of several kinds of vesicles to plasma membrane [8, 54, 55]. Third, secretory autophagy is enhanced by inhibition of autophagosome/lysosome fusion or impairment of lysosome function [8, 55].

Besides governing the secretion of autophagosomes/amphisomes, autophagic machinery mediates the secretion of another type of EVs and this process is called LC3-dependent extracellular vesicle loading and secretion (LDELS) [6]. In LDELS, LC3 is recruited to the limiting membrane of MVBs, where it is responsible for sorting a number of RNA-binding proteins and non-coding RNAs into the ILVs. Interestingly, LDELS does not require the upstream autophagic proteins such as FIP200 and ATG14. This indicates that the LC3 conjugation system facilitates the delivery of LC3 to endosome, rather than the formation of autophagosome. In addition, EVs formed by LDELS require neutral sphingomyelinase SMPD3 and its regulator NSMAF. Thus, LDELS shares certain similarities to exosome biogenesis.

#### Migrasomes

Migrasomes are formed on the retraction fibers of migrating cells and carry signaling molecules (cytokines, chemokines, and growth factors) and RNAs for intercellular communications [10]. Although the detailed biogenesis mechanism of migrasomes has not been completely understood, recent studies point out a three-stage migrasome formation mechanism: nucleation, maturation, and expansion. In addition, migrasome formation involves the coordination of biochemical and biophysical events. First, the nucleation occurs actually at the leading edge of migrating cells, where sphingomyelin synthase 2 clusters to form foci [57]. These foci mark migrasome formation sites (MFSs) and are stationary. Thus, when the cell moves forward, the MFSs are gradually located to the cell rear and finally concentrated at the junctions of retraction fibers. At the maturation stage, phosphatidylinositol phosphate 5-kinase (PIP5KIα) is recruited to MFSs to generate PI(4,5)P_2_, which in turns recruits Rab35 [58]. The active Rab35 subsequently binds to integrin, thereby enhancing the adhesion of MFSs to the extracellular matrix and stabilizing the retraction fiber junctions. The fluctuation of membrane tension in the retraction fiber junctions during cell migration also initiates membrane swelling at MFSs. Finally, at the stage of expansion, tetraspanin 4 (Tspan4) is recruited to the membrane swelling to assemble tetraspanin-enriched microdomains (TEMs). The continuous migration of TEMs towards the membrane swelling, together with the membrane-bending rigidity generated by Tspan4 and cholesterol in TEMs, leads to the enhancement of membrane swelling and the formation of tetraspanin-enriched macrodomains (TEMAs) and subsequently, the mature migrasomes [59, 60].

In summary, the biogenesis of apoptotic bodies shares certain similarities to that of large ectosomes, whereas the biogenesis of secretory autophagosomes/amphisomes is diverted from autophagy pathway. Migrasomes, however, exploit a unique, three-stage mechanism for their formation. The characters, markers, and biogenesis mechanisms of canonical and atypical EVs are summarized in Table 1.

**Table 1:** Characteristics of different EVs

Types of extracellular vesicles	Size (nm)	Biomarkers	Mechanism of formation	Biogenesis pathways	Intracellular origin	References
Exosomes	50–150	CD63, CD81. CD9, TSG101, ALIX, Flotillin, HSP70, HSP90, TSPAN29, TSPAN30	Multivesicular bodies fusion with plasma membrane	ESCRT-dependent, tetraspanin-dependent, ceramide-dependent, lipid rafts	Endosomes	[266]
Microvesicles	100–1000	Integrins, selectins, metalloproteinase	Outward shedding of the plasma membrane	ESCRT-dependent, Rho/ROCK/myosin pathway, I-BAR, membrane protrusion	Plasma membranes	[267]
Large ectosomes	> 1000	Phosphatidylserine	Outward shedding of the plasma membrane	Ca2 + -dependent, flippase/floppase	Plasma membranes	[268]
Apoptotic bodies	> 1000	Annexin V, phosphatidylserine	Cytoplasmic fragmentation during programmed cell death	ROCK, LIMK, pannexin 1, caspase 3	Cytoplasmic fragmentation	[269]
Secretory autophagosomes	300–900	LC3B, p62 dsDNA	Autophagosome fusion with plasma membrane	Cellular stress, lysosome inhibition	Endosomes	[270]
Migrasomes	500–3000	TSPAN4, TSPAN7, integrins	Retraction fiber break down at the cell rear during migration	Rab35, PIP5Kiα, TSPAN4	Migrating cells	[271]

#### Exosome secretion

Ectosomes and exosomes are released through distinct mechanisms. Ectosomes are secreted by fission from the plasma membrane, which is mainly mediated by actin cytoskeleton contractility or by ESCRT-III and VPS4 in the case of ARMMs, as described above. The secretion of exosomes, however, requires the transport of MVBs to cell surface and the fusion of MVBs with plasma membrane. Furthermore, exosome secretion requires the avoidance of lysosomal degradation of MVBs. Below, we highlight the important steps of exosome secretion and the mechanisms that determine the fate of MVBs between degradation and secretion.

#### MVB transport and fusion with plasma membrane

The long-range transport of MVBs from their formation sites to the cell surface is mediated by microtubule (MT) and MT-associated motor proteins. In particular, kinesin- or dynein-family motors are responsible for transporting MVBs towards cell surface or lysosomes, respectively. Rab7 loading on the MVBs membrane recruits dynein for targeting MVBs towards lysosomes at the expense of exosome secretion [61–63]. By contrast, Rab27b promotes the motility of MVBs from perinuclear region towards plasma membrane [64]. Once MVBs travel to the cell surface, the branched cortical actin facilitates the docking of MVBs to plasma membrane. While actin-binding protein cortactin stabilizes cortical actins to promote docking, coronin 1b antagonizes this function. Rab27a, however, inhibits the localization of coronin 1b to cortical actin, thereby enhancing MVBs docking [65]. Thus, the two Rab27 isoforms promote exosome secretion through distinct mechanisms [64]. Additionally, Rab35 and Rab11 are also implicated in MVBs-plasma membrane docking [66, 67]. The final step of exosome secretion is the fusion of MVBs limiting membrane with the plasma membrane, which involves different SNARE complexes and synaptotagmin-family members in a cell context-dependent manner [68–70].

#### Control of secretory vs. degradative fate of MVBs

MVBs are primarily destined to degradation by fusion with lysosomes. The choice between degradative and secretory fates of MVBs is not fully understood, but this undoubtedly affects cell physiology and function. In principle, molecules that regulate the MVBs transport and docking/fusion processes would affect this choice. For instance, a subclass of MVBs that is destined to exosome secretion undergoes a Rab7a/Arl8/Rab27a switch and the cholesterol sensing protein ORP1L at the ER-late endosome membrane contact sites regulates this switch [63]. In contrast to Rab7, Arl8b recruits kinesin and therefore facilitates the motility of MVBs towards cell surface. In addition, KIBRA stabilizes Rab27a by inhibiting its proteasomal degradation, thereby promoting exosome secretion [71]. The exocyst complex, which targets and tethers post-Golgi vesicles to plasma membrane during conventional secretion [72], also directs MVBs towards plasma membrane to promote the secretory fate of MVBs. Interestingly, a switch of phosphatidylinositol-3-phosphate to phosphatidylinositol-4-phosphate on the MVBs membrane facilitates the recruitment of exocyst complex, thereby promoting exosome secretion [73]. Accordingly, matrix stiffness stimulates exosome secretion through AKT-mediated activation of Rab8, an exocyst regulator [74].

In addition to the mechanisms that regulate MVBs transport and plasma membrane docking/fusion, evidence has emerged that MVBs biogenesis mechanism could influence on its degradative vs. secretory fate. For instance, ISGylation of ESCRT component such as TSG101 promotes the degradation of MVBs at the expense of exosome secretion [75]. Furthermore, MVBs formed by syndecan-syntenin-ALIX pathway seem to favor a secretory fate and the promotion of syndecan 4-syntenin lysosomal degradation by tetraspanin-6 inhibits exosome release [76]. Recently, direct evidence for a coupling between MVBs biogenesis mechanisms and their fate decision has been reported [77]. The two ESCRT-associated, Bro1-family proteins ALIX and PTPN23 (also known as HD-PTP) compete for binding to syntenin and therefore govern the formation of two types of MVBs. While the ALIX-containing MVBs are directed to plasma membrane for secretion, the PTPN23-containing MVBs undergo lysosome degradation. This finding is in line with a recent CRISPR-based screen, which identifies PTPN23 as a prominent inhibitor of sEV secretion [78]. Mechanistically, ALIX prevents the accumulation of branched actin filaments around MVBs by recruiting actin capping proteins, thereby promoting MVBs motility. Furthermore, ALIX- and PTPN23-containing MVBs recruit Arl8b and Rab7, further facilitating directional trafficking of MVBs towards plasma membrane and lysosomes, respectively. An intriguing concept is that the different molecules involving in the distinct MVB biogenesis mechanisms could recruit regulators of lysosome fusion, MVBs transport, docking or fusion, thereby impacting on MVBs’ fate decision. These different molecules also likely couple MVB biogenesis with the sorting of different cargos, thereby regulating the functions of exosomes.

#### Tumor immune microenvironment (TIME)

TIME is a complex ecosystem sustained by tumor cells, immune cells, stromal cells, the extracellular matrix, and blood vessels. It includes immune cells such as lymphocytes (T cells and B cells), natural killer (NK) cells, macrophages, dendritic cells (DCs), myeloid-derived suppressor cells (MDSCs), and neutrophils, as well as non-immune cells like cancer-associated fibroblasts (CAFs) [79]. Although the immune system is known as a robust defense mechanism in the human body, whether immune cells within the TIME promote or suppress tumor growth remains controversial. Pro-tumor elements, such as regulatory T cells (Tregs) and MDSCs, create an immunosuppressive environment, inhibiting anti-tumor immunity via cytokines (TGF-β and IL-10) and immune checkpoint molecules (PD-L1, CTLA-4, TIM-3, TIGIT, and NRP1) [80, 81]. Conversely, cytotoxic T cells destroy tumor cells through the secretion of granzyme and perforin, especially when activated by immune checkpoint blockade [82]. The TIME’s composition and dynamics influence tumor progression, migration, invasion, metastasis, and response to immunotherapy.

#### The effects of tumor-derived extracellular vesicles (TEVs) on TIME

TEVs are key modulators of the TIME, orchestrating immune evasion and tumor progression. TEVs, including microvesicles and exosomes, are known to reprogram immune cell functions. They suppress anti-tumor immunity by inhibiting CD8 + T cell cytotoxicity or promoting their exhaustion through upregulating immune checkpoint protein, reducing granzyme and perforin secretion, and inducing apoptosis [83]. TEVs also promote Treg expansion and stability to enhance immunosuppression via cytokine secretion [84]. Additionally, TEVs polarize macrophages towards a pro-tumor M2 phenotype, impair DC maturation, and reduce NK cell activity by downregulating natural killer group 2D (NKG2D) [85, 86]. They further drive MDSCs and neutrophil polarization for obtaining immunosuppressive properties [87, 88]. By modulating these immune cells, TEVs facilitate tumor growth, metastasis, and resistance to immunotherapy through the encapsulated cargos, thereby offering new insights as potential targets for clinical therapeutic intervention. Interestingly, the sorting of EV cargo such as immune modulatory molecule PD-L1 was reported to be controlled by ESCRT-0 component HRS by different groups [89, 90], implying the coupling of EV biogenesis and cargo sorting machinery. The detailed functional mechanisms of TEVs on TIME are described below.

#### The effects of TEVs on lymphocytes

##### *CD8* + *T Cells*

CD8 + T cells, also known as cytotoxic T cells, are a subset of T lymphocytes critical for adaptive immunity. They express the CD8 co-receptor, which binds to MHC class I molecules on the antigen presenting cells (APCs) to enable the recognition of specific antigens. Upon activation by antigen presentation and co-stimulatory signals (CD28-CD80/86), CD8 + T cells proliferate and differentiate into the effector cells that respond to infection or cancer cells by releasing cytotoxic molecules, such as granzyme and perforin [91]. Moreover, they also secrete cytokines like IFN-γ to sustain the expansion of memory T cells or to activate macrophages. In the TIME, CD8 + T cells play a pivotal role in anti-tumor immunity [92]. Interestingly, their anti-tumor function can be suppressed by TEVs through the modulation of proliferation, activation, immune checkpoint molecule expression and metabolic perturbation.

Nucleic acids, including microRNAs (miRNAs), circular RNAs (circRNAs), and long non-coding RNAs (lncRNAs), have been discovered as a major type of TEV cargos to significantly modulate CD8 + T cell function for promoting immune evasion. For instance, miR-424 carried by colorectal cancer (CRC)-derived EVs downregulates the expression of CD28 in T cells, thereby attenuating the CD28-CD80/86 costimulatory pathway and inhibiting T cell activation to facilitate immune evasion [93]. Additionally, circCCAR1, encapsulated within hepatocellular carcinoma (HCC)-derived EVs, is internalized by CD8 + T cells to stabilize PD-1 expression, thereby promoting resistance to anti-PD-1 immunotherapy [94]. CircRNA-002178 enriched in lung cancer-derived EVs upregulates PD-L1 and PD-1 expression in cancer cells and CD8 + T cells, respectively, leading to increased T cell exhaustion and diminished tumor clearance [95]. Furthermore, leukemia cell-derived EVs contain miR-19a-3p to inhibit the expression of creatine-specific transporter, SLC6A8 in CD8 + T cells. The reduction of creatine level reduces ATP generation in CD8 + T cell, thereby mitigating their cytotoxic function to facilitate leukemia cell immune evasion [96]. EVs secreted by gastric cancer contain miR-1246, which stabilizes PD-L1 on lymphatic endothelial cells and promotes CD8 + T cell apoptosis [97]. Several miRNAs, such as miR-3187-3p, miR-498, miR-122, miR-149, and miR-181a/b, are enriched in melanoma cell-derived EVs. They act together to inhibit T cell receptor (TCR) signaling and granzyme B secretion, thus reducing T cell cytotoxic activity [98]. Moreover, lung cancer-derived EVs are enriched with circUSP7 to attenuate granzyme B and perforin secretion by CD8 + T cells, thereby facilitating lung cancer resistance to anti-PD-1 immunotherapy [99]. Similarly, triple-negative breast cancer (TNBC)-derived EVs contain high amounts of circmiR-20a-5p to downregulate NPAT expression in CD8 + T cells, leading to T cell dysfunction and resistance to anti-PD-1 therapy [100].

Apart from RNAs, regulatory proteins that are preferentially sorted into TEVs alter the immune surveillance functions of CD8 + T cells. For example, BMI1 is packaged in cholangiocarcinoma-derived EVs to inhibit the secretion of chemokines responsible for CD8 + T cell recruitment [101]. Moreover, arginase-1-bearing EVs produced by ovarian carcinomas inhibit CD8 + T cell proliferation by downregulating CD3ζ [102]. Additionally, glioblastoma- and prostate cancer-derived EVs carry membrane-bound FasL, which interacts with Fas receptors on the CD8 + T cells surface to induce T cell apoptosis [103, 104]. Furthermore, active TGF-β type II receptor is transferred from breast cancer-derived EVs to CD8 + T cells to stimulate TGF-β signaling and CD8 + T cell exhaustion [105].

In addition to altering cytokine secretion and TCR signal transduction, TEVs also regulate immune checkpoint protein expression, or serve as a vehicle to transfer immune checkpoint protein from cancer cells to CD8 + T cells, leading to T cell exhaustion and resistance to immune checkpoint blockade. For instance, EVs secreted by esophageal carcinoma stem cells enriched with OGT are taken up by the neighboring CD8 + T cells to enhance PD-1 expression [106]. HLA-G secreted by TEVs upregulates the expression of immune checkpoint proteins, such as CTLA-4, PD-1 and TIM-3 on CD8 + T cells, leading to T cell exhaustion [107]. In melanoma, TNBC, and nasopharyngeal carcinoma (NPC), PD-L1 is selectively sorted into EVs to suppress the function, proliferation, and infiltration of CD8 + T cells, thereby mitigating anti-tumor immune responses [89, 90, 108–110]. Additionally, ICAM-1, an adhesion receptor was discovered co-exist on melanoma-derived exosomes with PD-L1 [111]. Mechanistically, Rab27A and ESCRT-0 component Hrs positively regulate the secretion of exosomes enriched in ICAM-1 and PD-L1. Upon the interaction of ICAM-1 on TEVs with its ligand, LFA-1 on CD8 + T cells, PD-L1 on TEVs sequentially interacts with PD-1 on T cells, thereby promoting PD-L1-mediated T cell exhaustion. Other immune checkpoint protein such as NRP1 is found in non-small cell lung cancer (NSCLC)- and melanoma-derived exosomes. These exosomes are originated from the ALIX-derived MVBs in the endocytic pathway, which occupied with NRP1 to promote CD8 + T cell exhaustion, subsequently facilitates tumor progression [77].

Metabolic balance is extremely important for maintaining T cell expansion and cytotoxic activity. Several studies identified that TEVs alter the production of metabolites to perturb T cell metabolism, leading to T cell dysfunction. For example, glioblastoma-derived EVs enriched with CD73 upregulate adenosine levels, which binds to the adenosine receptor 2A on the surface of CD8 + T cells to inhibit glycolysis and TCA cycle. These metabolic perturbations suppress T cell clonal proliferation, thereby reducing cancer clearance [112]. Moreover, ENPP1 carried by breast and lung cancer-derived EVs hydrolyzes cGAMP in CD8 + T cells, which dampens cGAS-STING pathway to reduce T cell infiltration to TME [113]. In addition, ENTPD2 in CRC-derived EVs facilitates the conversion of ATP to adenosine, thus inhibiting CD8 + T cell cytotoxic activity and subsequently reshaping an immunosuppressive TME [114].

##### *CD4* + *T Cells*

CD4 + T cells, also known as T helper cells or Th cells, are crucial in regulating the innate and adaptive immune systems. Upon activation, they differentiate into specific subsets (Th1, Th2, Th17, or Treg), which secrete distinct cytokines to regulate other immune cells, including B cells and CD8 + T cells [115]. Intriguingly, various studies have reported that TEVs interact with or are taken up by CD4 + T cells, thereby altering anti-tumor immunity [116]. For instance, NPC-derived EVs, decorated with membrane-bound galectin-9, a ligand of the membrane receptor Tim-3, induce Th1 cell apoptosis to mitigate anti-tumor T cell responses. Blockade of galectin-9 and Tim-3 using neutralizing antibodies reduces the interaction between TEVs and Th1 cells, thereby elevating anti-tumor T cell responses [117]. Furthermore, several studies reported that PD-L1-enriched TEVs promote cancer immune evasion by reducing the abundance of CD4 + T cells, suggesting a pivotal role of immune checkpoint protein-bearing TEVs in regulating CD4 + T cells [118–120]. Interestingly, although p53 is a tumor-suppressing protein, p53 mutants have been widely reported to promote cancer growth by inactivating innate immune signaling through cytokine production [121, 122]. p53 mutants carried by NSCLC-derived EVs are taken up by CD4 + T cells, where it downregulates glycolysis to suppress CD4 + T cell number and function, leading to tumor immune escape [123].

##### Treg cells

Apart from Th cells, naïve T cells differentiate into Tregs upon antigen stimulation in the presence of TGF-β [124]. Tregs are a subpopulation of CD4 + T cells with enriched FoxP3 expression. They mitigate anti-tumor immune responses through numerous immunosuppressive mechanisms, including secretion of immunosuppressive cytokines such as IL-10 and IL-35 [125].

Similar to Th cells, TEVs enhance anti-immune properties of Tregs to facilitate immunosuppressive TME formation. In 2009, galectin-3 was reported to repress TCR-mediated CD4 + T cell activation, thereby remodeling the immune landscape in the TME [126]. Building on this, a recent study suggests that TEVs regulate the biological properties of Tregs through galectin-3-dependent signal transduction. Galectin-3 binding protein (Gal3BP) is enriched on the surface of TNBC-derived EVs, which binds to galectin-3 secreted by TNBC cells to form a Gal3BP/Gal3 complex. This complex on the EV surface then interacts with CD45 on the Tregs surface to induce Treg secretion of IL-10 and IL-35 for immunosuppression [127]. In addition, NPC-derived EVs enriched with CCL20 chemokine promotes the conversion of CD4 + T cells into Tregs and facilitates the recruitment of Tregs to TME [128]. Moreover, cirGSE1, found in HCC-derived EVs, promotes Treg expansion by upregulating TGFβRI/Smad3 signaling pathway [129]. A recent study also revealed that melanoma-derived exosomes preferentially encapsulate NRP1 to enhance the recruitment of PD1 + and NRP1 + Tregs to TME, thereby promoting tumor growth [77].

Extracellular adenosine is derived from ATP through the sequential catalytic activities of membrane-associated CD39 and CD73 [130]. Importantly, CD39 in NSCLC-derived EVs hydrolyzes ATP to activate AMPK signaling in Tregs, thereby increasing FoxP3 expression to enhance Treg immunosuppressive function [131]. Similarly, EVs isolated from head and neck cancer and bladder cancer carry high levels of CD39 and CD73, which facilitate adenosine generation to support Tregs pro-tumor functions [132, 133].

##### γδ T Cells

The majority of T cells are αβ T cells, which express a heterodimeric TCR composed of α and β chains. Gamma delta (γδ) T cells are a distinct subset of T lymphocytes with TCRs composed of γ and δ chains. γδ T cells are typically enriched in peripheral tissues, such as mucosal and epithelial tissues, rather than secondary lymphoid organs [134]. Several studies revealed the tumor-promoting roles of TEVs in regulating γδ T cells signaling [135]. For example, lncRNA SNHG16 in breast cancer-derived EVs expands the population of CD73 + γδ T cells through the TGF-β1/Smad5 pathway, thereby boosting the production of pro-tumor metabolite and adenosine to promote tumor growth [136]. Additionally, gastric cancer-derived EVs enriched with miR-135b-5p are taken up by Vγ9Vδ2 T cells, a type of γδ T cells, leading to their apoptosis and reducing the production of cytokines like IFN-γ and TNF-α, thereby dampening cytotoxic T cell activation [137].

##### B cells

B cells, also known as B lymphocytes, are mononuclear lymphoid cells that function in antigen presentation and antibody production. B cells are important not only in adaptive immunity but also in regulating TME, alongside Tfh cells and other APCs [138]. Although B cells mainly exert anti-tumor effects, regulatory B cells (Bregs) possess immunosuppressive functions by secreting cytokines such as IL-10, IL-35, and TGF-β, together with adenosine and granzyme B [139].

To date, numerous studies have shown the impact of TEVs on B cells and/or Bregs. For instance, EVs derived from esophageal squamous cell carcinoma patients suppress B cell proliferation and promote the expansion of Bregs and PD-1^high^ Bregs, thereby reshaping an immunosuppressive TME [140]. HCC-derived EVs enriched with HMGB1 promote TIM-1 + Bregs expansion through upregulating toll-like receptor (TLR) 2/4 and MAPK signaling pathways [141]. Additionally, lncRNA HOTAIR, selectively encapsulated by CRC-derived EVs, enhances PD-L1 expression on B cells to suppress CD8 + T cell cytotoxic activity [142]. Furthermore, a comprehensive profiling of pancreatic ductal adenocarcinoma (PDAC)-derived EVs showed the existence of a diverse repertoire of tumor-associated antigens on the surface of EVs, which serve as decoys by binding to autoantibody generated by B cells, thereby allowing cancer cells to escape from complement-mediated cytotoxicity [143].

#### The effects of TEVs on macrophages

Macrophages are immune cells that play a crucial role in response to pathogens and foreign substances, including tumor cells. Their remarkable plasticity enables them to adapt to the complex microenvironment [144]. In cancer, tumor-associated macrophages (TAMs) undergo polarization into two major populations: M1 and M2 macrophages. Generally, M1 macrophages, activated by IFN-γ, LPS, or TNF-α, exhibit anti-tumor effects. Conversely, M2 macrophages, induced by IL-4, IL-13, IL-10, or TGF-β, play a pro-tumor role [145–147]. M2-like macrophage polarization is a hallmark of cancer progression, and recent studies have uncovered unique functions of TEVs in reshaping TAM plasticity and pathological functions, particularly in establishing an immunosuppressive TME.

Nucleic acids enriched in the TEVs are reported to induce macrophage polarization and pathological functions. For instance, CRC-derived EVs carry miR-21-5p and miR-200a to downregulate SOCS1 and PTEN in macrophages, respectively, thereby activating STAT1 and AKT signaling pathways to promote PD-L1 expression and macrophage M2-like polarization. Such PD-L1-enriched macrophages induce CD8 + T cell exhaustion, subsequently reducing T cell cytotoxic activity and promoting tumor progression [148]. Furthermore, oral squamous cell carcinoma-derived EVs enriched with lncRNA UCA1 reactivate LAMC2-mediated PI3K/AKT signaling in macrophages to promote their M2-like polarization [149]. Similarly, circATP2B4 in ovarian cancer-derived EVs rescues the expression of SREBF1, thus reviving the PI3K/AKT axis and promoting M2 macrophage polarization [150]. Additionally, piR-30937-containing TEVs inhibit PTEN and activate AKT signaling to promote the expression of CD276 in macrophages. The CD276 + macrophages in turn inhibit T cells proliferation and IFN-γ production, thus dampening anti-tumor immunity [151]. Moreover, glioblastoma cell-derived EVs enriched with miR-25/93 suppress cGAS-STING pathway in macrophages, thus downregulating M1 properties of TAMs to further attenuate T cells recruitment to TME [152].

Apart from nucleic acids, regulatory cytokines and immune checkpoint proteins that fuel the pro-tumor function of macrophages are also carried by TEVs. For example, CD133-containing TEVs promote M2-like polarization of TAMs and stimulate IL-6 secretion to activate STAT3 signaling, thereby accelerating CRC progression [153]. Moreover, gastric cancer-derived EVs stimulate TAMs to secrete excessive IL-10, which serves as an inhibitory cytokine to impair CD8 + T function, thereby sustaining tumor growth [154]. Additionally, GOLM1 stabilizes PD-L1 expression to promote the secretion of PD-L1-enriched TEVs from HCC. Such TEVs transfer PD-L1 to TAMs, which further represses CD8 + T cell activity [155]. Glioblastoma-derived EVs enriched with FasL and CTLA-4 activate the NF-κB pathway in macrophages, which promotes M2 macrophage polarization to reshape an immunosuppressive TME [102]. Recent study reported that CXCL1-containing TEVs activate PD-L1 signaling in TAMs, leading to TNBC metastasis [156]. Furthermore, laminin-enriched TEVs stimulate the integrin αvβ5/AKT/Sp1 signaling pathway in macrophage, thereby facilitating macrophage M2 polarization to promote tumor growth [157]. Interestingly, TEV-mediated metabolic regulation of macrophages plays indispensable roles in TME regulation as well. For instance, long-chain fatty acid-containing TEVs are taken up by macrophages to facilitate fatty acid oxidation. Such metabolic perturbation favors the conversion of M1 macrophages to an M2-like phenotype [158]. Furthermore, LDHA promotes the sorting of EPHA2 to clear cell renal cell carcinoma (ccRCC)-derived EVs, which facilitates M2-type macrophage polarization and promotes tumor immune evasion [159]. Gastric cancer-derived EVs with excessive amounts of ELFN1-AS1 upregulate PKM and GLUT1 expression in M1 macrophages, which promotes glucose uptake and lactate production. Such metabolic reprogramming accelerates the differentiation of M2 macrophage, thus promoting the formation of an immunosuppressive TME [160]. Interestingly, nicotinamide encapsulated in TEVs alters macrophage NAD + metabolism to activate IL-6/NF-κB signaling, thereby increasing the amount of pro-tumor macrophages in the TME [161].

Together, these studies demonstrate that TEVs contribute to the M1 to M2 polarization of macrophages. The reduction of M1-like macrophages dampens the anti-tumor immunity, while the increased of M2-like macrophages mitigates CD8 + T cells activity. Thus, understanding how TEVs regulate macrophage differentiation is crucial for future immunotherapies.

#### The effects of TEVs on CAFs

Fibroblasts and CAFs are cells primarily located in connective tissues. While fibroblasts are essential for healthy tissue maintenance, CAFs, a specialized subset located within or close to tumors, are reprogrammed by tumors to promote cancer progression [162].

Numerous studies have reported that CAFs are stimulated by TEVs to promote tumor progression and to modulate immune surveillance [163]. For example, ovarian cancer-derived EVs upregulate CAF markers such as α-SMA, vimentin, desmin, and FSP-1 to facilitate normal fibroblasts to CAF-like transformation, thereby upregulating their motility, proliferation, invasiveness, and the pro-tumor crosstalk between tumor cells and CAFs [164]. Similarly, TEVs enriched with telomerase upregulate the expression of α-SMA and vimentin in fibroblasts, which facilitates the transformation of CAFs [165]. Additionally, TEVs induce the IL-6 and IL-17 signaling in CAFs, which promotes the secretion of pro-inflammatory cytokines in TME, thereby contributing to the progression of tumor malignancies [166].

Apart from facilitating CAFs polarization, TEVs can activate CAFs to reprogram immune cells in TIME. For example, CRC-derived EVs enriched with TGF-β1 are taken up by hepatic stellate cells, a type of liver fibroblast, thus promoting their transformation into CAFs. These activated CAFs downregulate NKG2D expression in NK cells to inhibit their tumor-killing ability and recruit MDSCs, thereby creating an immunosuppressive TIME to promote liver metastasis [167]. Furthermore, Lewis lung carcinoma-derived EVs stimulate lung fibroblasts to secrete excessive amounts of CCL1, an inflammatory cytokine, thereby activating CCL1-CCR8 axis in Tregs to promote their differentiation and pro-tumor function [168].

RNAs carried by TEVs also play significant roles in modulating the phenotype and function of CAFs. For instance, recent study reported that SUR1 reduces the loading of let-7a-5p to NSCLC-derived EVs. As let-7a-5p downregulates TGFβRI, the reduction of let-7a-5p reactivates the TGF-β pathway in fibroblasts, thus promoting their transformation into CAFs [169]. Overall, these studies highlight the critical role of TEVs in stimulating CAF transformation and empowering CAFs to reprogram other cell populations, subsequently shaping an immunosuppressive TME to promote malignancies.

#### The effects of TEVs on DCs

DCs are professional APCs that play an important role in presenting antigens to T cells for maintaining anti-tumor immunity [170]. However, immunosuppressive TME may alter DC function, phenotype, and tolerogenicity through cytokines, direct cell–cell interactions, and EV-mediated signal transduction, thereby mitigating DCs’ anti-tumor efficacy [171].

Several studies have reported the regulatory role of TEVs in suppressing DC anti-tumor functions. For example, galectin-9 on glioblastoma-derived EVs binds TIM-3 receptor on DCs, which inhibits DC antigen presentation and suppresses T cell-mediated anti-tumor immune responses [172, 173]. Similarly, NPC-derived EVs enriched with galectin-9 impair DC oxidative phosphorylation, thereby reducing their capacity in activating cytotoxic T cells [174]. Additionally, melanoma-derived EVs enriched with HSP72 and HSP105 activate TLR2 and TLR4 on DCs to promote IL-6 production. The excessive IL-6 secreted by DCs stimulates STAT3 signaling in melanoma to upregulate MMP9 expression, thereby promoting tumor cell invasion [175]. Prostate cancer-derived EVs with PGE2 induce the expression of DC surface enzyme CD73 to hydrolyze AMP to adenosine, which inhibits CD8 + T cell activity [176].

DC maturation is a critical process for acquiring the ability to present antigen to T cells. Several reports revealed that cancer cells modulate DC maturation through TEVs (177)For instance, melanoma-derived EVs enriched with S100A9 downregulate the expression of DC costimulatory molecules, CD83 and CD86, which mitigates DC maturation and subsequently delays T cell activation [178, 179]. Additionally, Lewis lung carcinoma- and 4T1-derived EVs block the differentiation of myeloid precursor cells into DCs and reduce the expression of DC chemokine receptors such as CCR6, CCR7 and CXCR3. These immature DCs lack the ability to prime Th1 cells, thereby dampening T cell-mediated anti-tumor activity [180].

Interestingly, EV-mediated metabolic perturbation is also a key factor leading to DC dysfunction. For example, fatty acids encapsulated by TEVs are delivered to DCs to stimulate lipid droplet biogenesis and to enhance fatty acid oxidation through PPARα, which reprograms DC metabolism toward mitochondrial oxidative phosphorylation, subsequently leading to DC dysfunction and tumor immune evasion [181, 182]

#### The effects of TEVs on NK cells

NK cells are immune cells that rapidly respond to tumors and play a critical role in eliminating cancer cells [183]. Unlike other immune cells, NK cells are able to clear pathogens through activating/inhibitory receptors without prior antigen sensitization. To date, several studies revealed that TEVs reshape the biological properties of NK cells, including proliferation, cytotoxic activity, cytokine secretion, metabolism, and viability [184].

The NK cell-activating receptor, NKG2D, detects stress-induced ligands (MICA and MICB) or pathogen-associated molecules on compromised cells. Upon binding of NKG2D to these ligands, NK cells release granzyme and perforin to mediate tumor cytotoxic lysis and elimination [185–190]. It has been reported that TEVs enriched with MICA, MICB and ULBP downregulate NKG2D expression on NK cells, thus reducing NK cell-mediated tumor clearance [191–194]. Align with these studies, glioblastoma-derived EVs enriched with immunosuppressive molecules, including FasL, CTLA-4, CD39, and CD73 suppress the expression of NKG2D in NK cells, thereby mitigating NK cell activation [102]. Similar to other immune cells, TGF-β signaling plays an indispensable role in suppressing NK cell anti-tumor function. TGF-β1 carried by ccRCC-derived EVs activates the TGF-β/Smad signaling in NK cells, thereby impairing their cytotoxic function to result in tumor immune evasion [195]. Recent study reported that acute myeloid leukemia-derived EVs decorated with PD-L1 suppress NK cell activation and cytotoxicity [196–198]. Moreover, the downregulation of FBP1 reduces the expression and loading of PKLR, a pyruvate kinase, to HCC-derived EVs. These PKLR-deficient TEVs disrupt NK cell metabolic balance to potentiate tumorigenesis [199].

In addition to protein cargos, RNA cargos in TEVs also play a critical role in regulating NK cell tumor-killing capability. For instance, HCC-derived EVs carry miR-17-5p to downregulate RUNX1, thus limiting the expression of its transcriptional target NKG2D in NK cell to result in NK cell dysfunction [200]. Additionally, miR-92b enriched in HCC-derived EVs downregulates NK cell stimulatory receptor, CD69 to inactivate NK cells [201]. Moreover, HCC-derived EVs with circUHRF1 promotes TIM-3 expression on NK cells to drive NK cell dysfunction, thereby promoting resistance to immunotherapy [202]. In bladder cancer, miR-221-5p and miR-186-5p are found in TEVs, which disrupt the stability of CD96 and perforin genes in NK cells to reduce their cytotoxic activity [203]. Furthermore, lung cancer-derived EVs enriched with miR-150-5p suppress the expression of NK cell activating receptor, CD226. These inactivated NK cells secrete pro-tumor molecules to induce angiogenesis and inflammation, thereby accelerating lung cancer tumorigenesis [204]. Moreover, lncRNA SNHG10 in CRC-derived EVs upregulates INHBC in NK cells to suppress the secretion of perforin and granzyme B, thereby reducing NK cell cytotoxic activity [205].

#### The effects of TEVs on MDSCs

MDSCs are a heterogeneous population of immature myeloid cells with immunosuppressive effects on TIME. Although MDSCs and DCs are differentiated from the same origin, unlike DCs, MDSCs suppress immune responses and protect tumors from immune attack [206, 207].

A number of studies have reported that TEVs carry RNAs to educate MDSCs, leading to increased tumor progression and immune evasion [208, 209]. For example, breast cancer-derived EVs enriched with miR-9 and miR-181a activate JAK/STAT signaling by downregulating SOCS3 and PIAS3, respectively, thereby expanding the amount of early-stage MDSCs for immunosuppression [210]. Moreover, melanoma-derived EVs bearing miR-125a-5p activate NF-κB signaling in MDSCs to upregulate PD-L1 expression, thus impairing anti-tumor response [211]. Additionally, oral squamous cell carcinoma-derived EVs enriched with miR-21 inhibit the expression of PTEN in MDSCs, thereby upregulating PD-L1 expression and subsequently inhibiting γδ T cell activity [212]. Similarly, gastric cancer-derived EVs encapsulating miR-107 suppress DICER1 and PTEN expression in MDSCs to activate PI3K signaling, thus elevating ARG1 expression to stimulate MDSCs expansion [213]. Furthermore, miR-10a and miR-21, selectively loaded into glioma-derived EVs under hypoxic environment, downregulate RORA and PTEN in MDSCs to maintain their differentiation and activation [214]. Additionally, PDAC-derived EVs deprived of miR-494-3p and miR-1260a promote calcium flux and glycolysis in MDSCs to facilitate their expansion [215]. Moreover, circRNA_0013936 encapsulated in bladder cancer-derived EVs upregulates FATP2 and downregulates RIPK3 in MDSCs to support their immunosuppressive function, subsequently suppressing CD8 + T cell activity [216].

Several studies also highlight the importance of protein cargos in TEVs in regulating MDSC immunosuppressive function [88]. For example, LILRB2, an inhibitory molecule, is encapsulated in glioblastoma-derived EVs to boost the expansion of MDSCs, thereby inhibiting CD8 + T cell proliferation [217]. Angiogenic factors such as VEGF are highly enriched in melanoma-derived EVs to promote MDSCs expansion for modulating an immunosuppressive TME [218]. Moreover, renal cancer- and CRC-derived EVs enriched with chaperone proteins like HSP70 and HSP60 activate MDSCs in TLR-dependent manner, thereby suppressing T cell functions [219, 220]. MIF in pancreatic cancer-derived EVs facilitates MDSC differentiation, recruitment, and activation, which in turn causes T cell dysfunction [221]. Together, these studies conclude that TEVs carry RNAs and proteins to reprogram MDSC biological functions by promoting their differentiation and immunosuppressive function.

#### The effects of TEVs on neutrophils

Neutrophils, the most abundant subset of granulocytes and immune cells in humans, play a pivotal role in the innate immune system against infection and cancer [222]. Neutrophils have bifunctional roles in cancer. They enhance the immune surveillance by secreting cytokines like IL-12 and TNF-α, which recruit and activate T cells to eliminate tumor cells. Conversely, neutrophils in the TME are polarized into pro-tumor N2-like neutrophils in response to TGF-β secreted by cancer cells [223]. Several studies revealed the function of TEVs in promoting neutrophil polarization and sustaining their pro-tumor functions. For example, melanoma-derived EVs promote the polarization of N2-like neutrophils by upregulating N2 molecular markers, such as ARG1, CXCR4, and VEGF, thereby mitigating the cytotoxicity of neutrophils to form an immunosuppressive TME [224]. Moreover, breast cancer-derived EVs induce N2 neutrophils to secrete IL-8, VEGF, and MMP9, thereby facilitating cancer progression [225]. Similarly, gastric cancer-derived EVs enriched with HMGB1 interact with TLR4 in neutrophils to activate NF-κB signaling, thereby activating the formation of immunosuppressive neutrophils [226]. Additionally, HMGB1 encapsulated by gastric cancer-derived EVs activates STAT3 signaling in neutrophils to upregulate PD-L1 expression, which suppresses T cell proliferation, activation and anti-tumor functions [227]. Furthermore, CRC-derived EVs carrying KRAS mutants stimulate neutrophil extracellular trap formation and IL-8 production, leading to CRC deterioration [228]. Moreover, circPACRGL encapsulated in CRC-derived EVs serves as a sponge for miR-142-3p and miR-506-3p to sustain TGF-β1 expression, thereby facilitating the differentiation of N1 to N2 neutrophils [229]. Additionally, head and neck squamous cell carcinoma-derived EVs enriched with PD-L1 and CD73 are taken up by neutrophils. These two proteins play synergistic role in mitigating T cell anti-tumor function, thereby facilitating tumor growth [230].

#### The effects of immune cell-derived EVs on TME

Although the roles of EVs derived from cancer cells are well-understood in EV biology, EVs secreted from various subsets of immune cells also play crucial role in shaping TME. For example, CD4 + T cell-derived EVs enriched with pro-inflammatory cytokines such as IFNγ, TNFα and IL-2, are taken up by macrophages to sustain M1 anti-tumor phenotype and to stimulate cGAS-independent STING activation, subsequently inhibiting CRC progression [231]. In addition, CD8 + T cell-derived EVs contain miR-298-5p, FasL, TNF-α, and PD-L1 to reduce the formation of mesenchymal tumor stromal cells including mesenchymal stem cells and CAFs, thereby mitigating melanoma progression [232]. Interestingly, compared to DC, DC-derived EVs are more proficient to activate CD8 + T cell and reduce breast cancer formation, presumably due to the highly concentrated MHC-I molecules on the EV surface, which is important for antigen presentation [233]. Furthermore, neutrophil-derived EVs preloaded with doxorubicin trigger caspase signaling pathway in CRC, thereby inhibiting CRC proliferation and promoting apoptosis [234]. M1 macrophage-derived EVs containing miR-150 mitigate glioma progression by directly downregulating MMP16 expression [235]. Moreover, under the stimulation of IL-15 and IL-21, NK cell-derived EVs preferentially sort CD226 to enhance cancer-killing ability [236].

Rather than inhibiting tumor progression, EVs secreted by certain subsets of immune cells promote tumor growth by promoting immune evasion. For example, MDSC-derived EVs enriched with TGF-β1 are speculated to induce the activation of Treg or Th17 cells, thereby forming an immunosuppressive TME [237]. CAF-derived EVs with excessive SNHG3 can be taken up by CRC to upregulate HuR expression and enhance HOXC6 transcription, leading to CRC proliferation [238]. Furthermore, M2 macrophage-derived EVs enriched with circ_CCDC66 restore the expression of MTDH, thereby promoting PD-L1 expression and subsequently mitigating CD8 + T cell cytotoxicity [239].

#### The effects of atypical EVs on TIME

Apart from typical EVs, several atypical EVs have been reported to modulate the TIME, including apoptotic bodies, migrasomes, and secretory autophagosomes. For instance, apoptotic bodies secreted by lymphoma cells inhibit CD8 + T cell and DC antitumor immunity [240]. Mechanistically, irradiation promotes the nuclear translocation of the transcription factor NF-AT to upregulate TGF-β1 expression. The immunosuppressive TGF-β1 then localizes to the membrane of apoptotic bodies secreted by lymphoma cells, thereby inhibiting the antitumor function of CD8 + T cells and DCs. Furthermore, apoptotic bodies secreted by murine leukemia cells promote the expression of chemokine receptor CCR7 in DCs to facilitate DC migration towards the lymph node for antigen presentation. These stimulated DCs induce T cell proliferation and IFNγ production, providing a potential antitumor function [241]. Interestingly, in CD169-/- mouse model, lymphoma-derived apoptotic bodies are taken up by DCs to enhance CD8 + T cells antitumor response, presumably due to the absence of immunosuppressive CD169 + macrophages [242]. In breast cancer cells, PD-L1 recruits β4 integrin at the cell rear to engage with cytoskeleton remodeling and RhoA activation, thereby facilitating rear retraction and supporting the release of PD-L1-containing migrasomes, which likely dampen antitumor immunity [243]. In addition, pancreatic cancer-derived migrasomes enriched in CXCL5, TGF-β1, ITGA2, Rab2b, and Rab32 are directly taken up by macrophages. These chemokines, cytokines and signaling molecules enriched migrasomes upregulate the expression of immunosuppressive factor ARG1, thus promoting macrophage M2 polarization to inhibit T cell proliferation and activation [244]. Similarly, autophagosomes secreted by melanoma cells stimulate p38-STAT3 signaling in macrophages through TLR4, thus promoting macrophages M2 polarization to inhibit CD8 + T cell antitumor functions [245]. Moreover, human liver cancer-derived LC3B + secretory autophagosomes are taken up by CD8 + T cells, which promote the expression of inhibitory receptor PD1, subsequently dampening the tumor killing ability of T cells [246].

In conclusion, atypical EVs such as migrasomes and secretory autophagosomes are derived from biogenesis pathways that differ from the canonical EVs such as exosomes and ectosomes, while apoptotic bodies are derived from unique cell status, i.e., apoptosis. Similar to canonical TEVs, these atypical EVs can carry immune-modulatory cargos to influence on TIME. However, different from the canonical TEVs, atypical EVs derived from cancer cells can elicit immunosuppressive or immune-stimulatory functions depending on the context of donor cells.

#### Applications of EVs in cancer diagnosis

EVs have emerged as a revolutionary tool in cancer diagnosis due to their ability to carry molecular cargos such as miRNAs and proteins, reflecting their tumor cell origins. Their stability in body fluids, protected by a lipid bilayer, makes them a powerful tool for non-invasive cancer diagnosis. Importantly, EVs offer a dynamic approach to capture tumor heterogeneity, surpassing the limitations of traditional tissue biopsies, which often miss the molecular variations within tumors [247].

Unlike tissue biopsies, which require invasive procedures and may not fully represent tumor heterogeneity, EVs provide a comprehensive molecular snapshot of the tumor microenvironment. Exosomal miRNAs, such as miR-21 and miR-1246, have been identified as reliable biomarkers for early-stage breast cancer detection [248]. Similarly, EV-associated proteins like CA-125, EpCAM, and CD24 are linked to ovarian cancer [249], offering potential for identifying specific cancer types. The stability of EVs in body fluids ensures that their cargos remain intact, making them ideal for repeated sampling to monitor disease progression or recurrence, unlike conventional imaging methods like computed tomography (CT), which detect only single tumor types and involve radiation exposure.

Recent technological advancements have significantly enhanced the ability to profile EVs for cancer diagnosis. For instance, Surface-Enhanced Raman Scattering (SERS) enable high-sensitivity and multiplexed detection of EV biomarkers at low concentrations [250]. This method allows precise identification of tumor-specific molecular signatures, improving early detection capabilities. For example, SERS can detect subtle differences in exosomal miRNA or protein profiles, distinguishing cancerous from non-cancerous states [251]. In addition, the integration of artificial intelligence (AI) with EV profiling further refines diagnostic accuracy. AI-driven analysis of SERS data identifies complex patterns in exosomal cargos, enabling simultaneous detection of multiple early-stage cancers, including lung, breast, colon, liver, pancreas and stomach [252]. This approach addresses the limitations of traditional diagnostics, which often require additional invasive tests and fail to capture tumor heterogeneity. These technologies collectively enhance the sensitivity and specificity of EV-based diagnostics, making them viable for clinical use to improve patient outcomes, ultimately revolutionize cancer diagnostics.

As of the current clinical cancer diagnosis, two EV-based liquid biopsy tests are commercially available for cancer detection. ExoDx™ analyzes urinary exosomal RNA to provide a non-invasive risk assessment for high-risk prostate cancer, offering high sensitivity in detecting clinically significant cases and reducing unnecessary biopsies compared to traditional Prostate-Specific Antigen testing [253]. In addition, Guardant360® platform profiles 55 cancer-related genes from circulating tumor DNA and EVs in blood, enabling comprehensive genomic and EV cargo insights across multiple solid tumors, including lung and breast cancer, with high specificity for guiding targeted therapies [254]. These two tests capitalize on the stability of EVs in body fluids, allowing repeatable, non-invasive sampling to monitor disease progression and treatment response, surpassing the limitations of invasive tissue biopsies and providing a dynamic, real-time view of tumor molecular signatures. The common biomarkers in EVs for cancer diagnosis are summarized in Table 2.

**Table 2:** Cancer biomarkers in EVs for diagnosis and prognosis

Cancer type	Sample type	Biomarkers	Application	References
Glioblastoma	Serum	EGFR, EGFRvIII	Diagnosis and prognosis	[272]
Colon cancer	Serum	Let-7a, miR-1229, miR-1246, CD147, CD9	Diagnosis	[273, 274]
Ovarian cancer	Plasma	CA0125, EpCAM, CD24	Diagnosis	[249]
Gastric cancer	Plasma	ARG1, CD3, PD-L1, PD-L2	Diagnosis and prognosis	[275]
Prostate cancer	Serum, urine	miR-107, miR-375, PCA-3	Diagnosis	[276, 277]
Brain cancer	Serum, cerebrospinal fluid	miR-320, miR-574-3p, miR-21	Diagnosis	[278, 279]
Breast cancer	Plasma	Developmental endothelial locus-1 (Del-1), miR-21, miR-1246, miR-200a/c, miR-205	Diagnosis and prognosis	[248, 280, 281]
Lung cancer	Pleural effusion, serum	miR-200b/c, miR-141, miR-375, miR-151a, miR-301a-3p, miR-629, miR-154-3p, miR-223, lncRNAs RP11-77G23.5, PHEX-AS1	Diagnosis	[282–285]
Pancreatic cancer	Serum	miR-17-5p, miR-1246, miR-4644, miR-4306, miR-3976, miR-21, mutated KRAS and p53 DNA	Diagnosis	[286–288]
Papillary thyroid carcinoma	Serum, plasma	miR-29a, miR-25-3p, miR-296-5p, miR-92a-3p, miR-5189-3p, miR-16–2-3p, miR-223-5p, miR-346, miR-34a-5p	Diagnosis and prognosis	[289–293]

While EVs hold significant promise for cancer diagnosis due to their molecular cargo and non-invasive nature, several limitations in their diagnostic sensitivity and specificity remain. The heterogeneity of EVs, while advantageous for capturing tumor diversity, can complicate the identification of consistent biomarkers across patients. For example, miR-21 is elevated in breast cancer [248], in the meantime, was found to be induced in cardiac disease [255]. The common biomarkers across multiple diseases can reduce cancer diagnostic specificity, leading to potential false positives or negatives. Additionally, advanced techniques like SERS require further optimization to consistently achieve high sensitivity at low EV concentrations, particularly for early-stage cancers where biomarker levels may be minimal. Furthermore, the reliance on AI-driven analysis, while enhancing pattern recognition, introduces challenges related to the need for large, well-annotated datasets to train models effectively, which may not always be available. These factors collectively hinder the standardization and widespread clinical adoption of EV-based diagnostics, necessitating further research to improve their sensitivity and specificity for reliable cancer detection.

#### EVs as a therapeutic strategy in TIME

In the context of the TIME, EVs have emerged as a promising therapeutic tools due to their ability to modulate immune responses, deliver therapeutic payloads, and influence tumor-immune interactions. To date, two main approaches have been explored for EV-based therapy: 1) inhibiting EV biogenesis and cargo sorting, 2) utilizing EVs as drug delivery vehicles.

#### Targeting TEV production and cargo loading

Given the profound and broad effects of TEVs on reshaping an immunosuppressive TME, blockage of their generation could be exploited as an anti-cancer strategy. Yeat et al. reported that cancer cells hijack syntenin, a key protein involved in exosome biogenesis, to promote exosome secretion and sorting of pro-tumor cargo, thereby facilitating lung cancer metastasis and tumor immune evasion [77]. Mechanistically, the tumor-promoting WDR4 acts as an E3 ligase substrate adaptor to trigger PTPN23 proteasomal degradation, which diverts the fate of MVBs towards exosome secretion. Such exosomes load with NRP1 and other pro-tumor proteins, such as MCAM and PTK2. Notably, a PTPN23-derived short peptide that blocks the interaction between WDR4 and PTPN23 [256] enables PTPN23 stabilization to promote MVB degradation in tumor cells, thereby reducing exosome secretion. In vivo studies also proved that this peptide inhibits tumor progression and lung metastasis, thus representing a potential therapeutic approach for further clinical validation.

Additionally, MAPK-mediated phosphorylation of HRS, an ESCT-0 component, not only promotes melanoma-derived exosome secretion, but selectively sorts PD-L1 into these exosomes, thus suppressing CD8 + T cell activation and their tumor infiltration ability [109]. It has been demonstrated that a phosphorylation-defective mutation on HRS reduces PD-L1 exosomal cargo loading and exosome secretion, thereby rejuvenating the anti-tumor function of CD8 + T cells. In principle, MAPK inhibitors may elicit similar effects, but a robust validation is needed to draw a solid conclusion.

CD47 expressed on the surface of tumor cells releases a “don’t eat me” signal to prevent tumor cells from the immune attack. Mechanistically, the binding between CD47 on tumor cells and SIRPα on DCs and macrophages mitigates phagocytosis activities of DCs and macrophages and further dampen their antigen presentation activity to result in tumor immune evasion [257]. Koh et al. showed that the engineered EVs harboring SIRPα variants (termed SIRPα-exosomes), can be used to competitively bind CD47 on tumor with macrophage, reviving the endocytosis of macrophages on tumor cells, thereby eliminating tumor cell in vitro and in vivo [258]. Thus, by inhibiting EV biogenesis or manipulating cargo loading, it is possible to reactivate the immune system to specifically target cancer cells.

#### EVs as drug delivery vehicles

Compared with conventional liposomes, EVs are widely recognized for their higher biocompatibility in treating cancer patients, due to their origin from the cellular endocytic pathway. Currently, many EV-based clinical trials targeting cancer progression can be found from ClinicalTrials.gov, which are summarized in Table 3. Below, we describe few representative trials.

**Table 3:** EV-based cancer therapy under clinical investigation

Clinical stage	EVs source	Cancer type	Treatment strategy	Status	Trial number
I	Plant-derived EVs	Colon cancer	Deliver curcumin	Recruiting	NCT01294072
I	Grape-derived EVs	Head and neck tumors (prevent oral mucositis)	Deliver grape extract	Completed	NCT01668849
I	Umbilical cord derived mesenchymal stem cell-derived EVs	Acute myeloid leukemia	Unmodified umbilical cord derived mesenchymal stem cell-derived EVs	Recruiting	NCT06245746
I	Mesenchymal stromal cell-derived EVs	Pancreatic cancer	Deliver KrasG12D siRNA	Active, not recruiting	NCT03608631
I	HEK293-derived EVs	HCC, liver metastases from primary gastric cancer or CRC	Deliver ASO-STAT6	Terminated	NCT05375604
I	DC-, macrophage- and tumor-derived EVs	Bladder cancer	Antitumor vaccine	Unknown	NCT05559177
II	DC-derived EVs	NSCLC	Antitumor vaccine	Completed	NCT01159288
II	DC-derived EVs	Melanoma	Antitumor vaccine	Suspended	NCT00042497

Curcumin, a polyphenol extracted from the turmeric plant, has been reported to trigger anti-inflammatory and anti-cancer signaling pathways, leading to the downregulation of oncogenic proteins such as NF-κB, JNK, and STAT3 [259]. In Phase I clinical trial NCT01294072, plant-derived EVs are used to deliver curcumin orally to treat colon cancer patients, aiming to downregulate oncogenic signaling and reactivate immune cells. Additionally, Phase I study NCT01668849 uses grape-derived EVs to treat patients who are undergoing chemoradiation for head and neck cancer, presumably to prevent oral mucositis.

Furthermore, Phase I study NCT03608631 uses engineered mesenchymal stromal cell-derived EVs enriched with KrasG12D siRNA to target pancreatic cancer, especially for metastatic PDAC patients. Similarly, Phase I study NCT06245746 employs mesenchymal stem cell-derived EVs to mitigate chemotherapy-induced myelosuppression in acute myeloid leukemia patients, aiming to reduce complications such as infections.

Phase II clinical trial (NCT01159288) investigates the use of DC-derived EVs as a maintenance immunotherapy following first-line chemotherapy in patients with NSCLC [260]. This trial evaluates the feasibility, safety, and immunological effects of IFN-γ-loaded DC-derived EVs in 22 patients with advanced (stage IIIB-IV) NSCLC who had stabilized or responded to chemotherapy. The primary endpoint was to achieve progression-free survival (PFS) in at least 50% of patients at four months post-chemotherapy, with secondary objectives including overall survival (OS), objective response rates, and biomarker analysis, particularly focusing on NK cell and T cell responses. In this trial, DC-derived EVs were produced from monocytes of HLA-A2 + patients, differentiated into DCs, and matured with IFN-γ to enhance MHC-II and BAG6 expression, the latter being a ligand for the NKp30 receptor. These EVs were loaded with tumor-associated antigens such as MAGE1, MAGE3 and NY-ESO-1 to stimulate tumor-associated antigen-specific immune responses. As anticipated, these DC-derived EVs significantly enhanced NKp30-dependent NK cell functions, particularly in patients with longer PFS (> 2.2 months). In addition, NKp30 cross-linking increased IFN-γ and TNF-α secretion, and CD107a upregulation, indicating NK cell activation, especially in patients with higher PFS. This study concludes that DC-derived EVs serve as a feasible and safe immunotherapy that preferentially boosts NK cell activity over T cell responses in NSCLC patients. The correlation between MHC-II, BAG6, and NKp30 activation highlights the potential of DC-derived EVs as an NK cell-targeted therapy, particularly for patients with advanced NSCLC, though further optimization is needed to enhance T cell responses and clinical efficacy.

#### Challenges of EV-based cancer therapy

In reality, EV-based cancer therapies are yet to be approved for clinical use, due to several significant challenges. Large-scale manufacturing is a primary hurdle [261]. High-quality EVs preparation requires scalable and reproducible processes while maintaining biological potency, which is complicated by the complex biogenesis mechanisms of EVs and the need for high-yield purification methods like ultracentrifugation. In addition, EV heterogeneity, stemming from variations in size, cargos, and surface markers due to donor cell variability or production conditions [34], further complicates the achievement of a uniform therapeutic efficacy and safety profiles. Furthermore, current standardized assays and benchmarks for the quality control of EVs are mostly designed for the use of laboratory research development, making it challenging to ensure batch-to-batch consistency in large-scale manufacturing. Importantly, regulatory compliance adds another layer of complexity. As agencies like the FDA require stringent guidelines for EV-based products, including detailed documentation of manufacturing processes, safety data, and clinical endpoints, which can be difficult to meet given the novel nature of EV therapeutics and the lack of established regulatory frameworks. These challenges collectively impede the translation of EV therapies from bench to bedside, requiring interdisciplinary efforts to advance manufacturing technologies, standardization protocols, and regulatory pathways.

## Conclusions

EVs, a group of nanoscale vesicles secreted by almost all cell types, were originally thought as “unwanted cellular disposals” [262]. With the tremendous amount of EV studies reported in the past few decades, scientist start noticing the importance of EVs in regulating physiological and pathological functions [263]. It is amazing that these tiny vesicles can accommodate enormous number of biomolecules including proteins, DNAs, RNAs, lipids and metabolites, which serve as critical mediators in the complex interplay between tumor cells and the immune system within the TIME [264]. In this review, we mainly discuss the biogenesis of EVs and their multifaceted roles in modulating immune responses to facilitate tumor progression, immune evasion, and metastasis. Additionally, we highlight the biogenesis and secretion mechanisms of various EV subtypes, underscoring their heterogeneity and functional diversity. The therapeutic potential of EVs, either by inhibiting their biogenesis or leveraging them as drug delivery vehicles, offers promising avenues for advancing cancer immunotherapy.

**Fig. 1:**
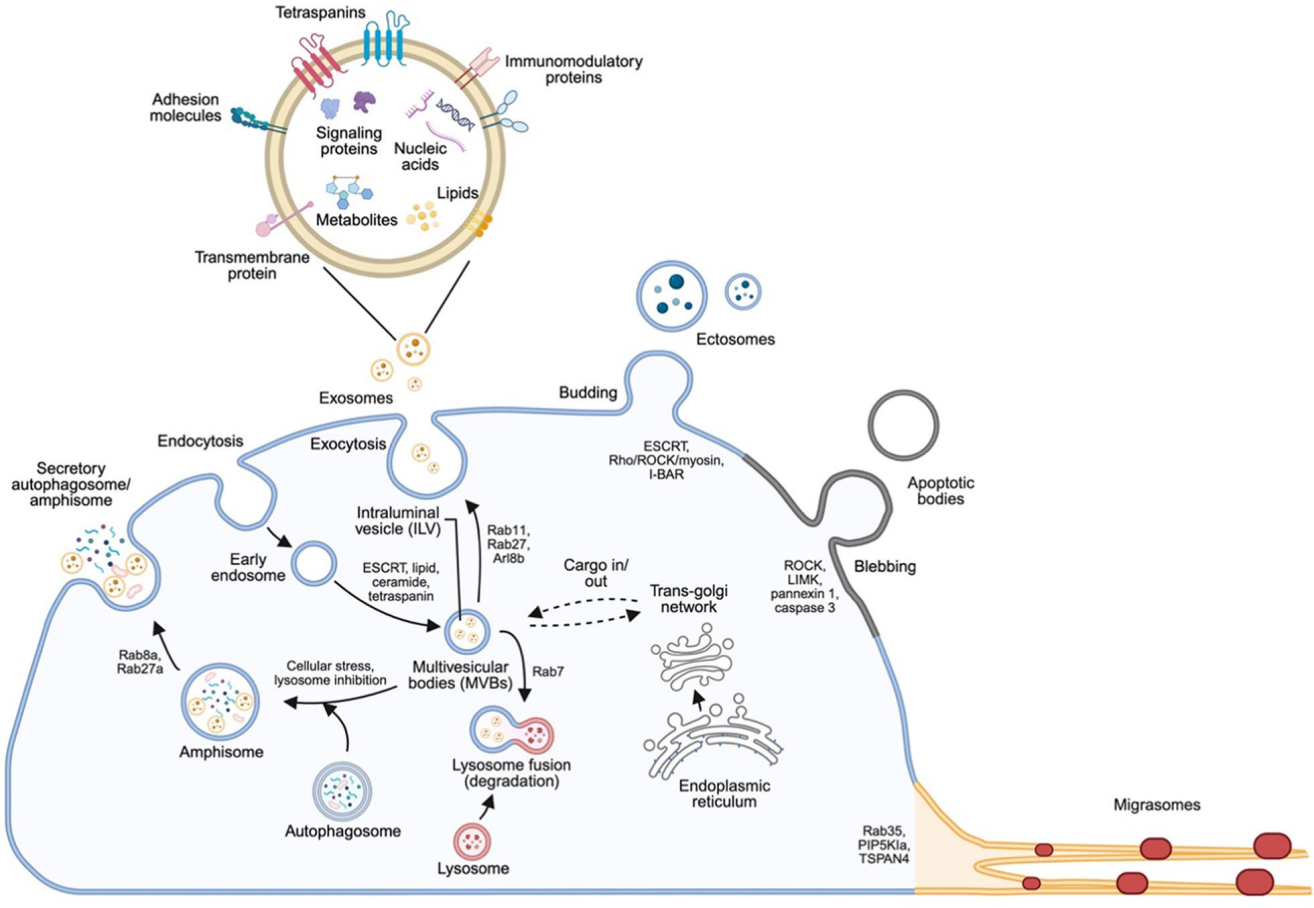
Mechanisms of EV biogenesis and EV components. EVs, including exosomes, ectosomes, apoptotic bodies, secretory autophagosomes/amphisomes and migrasomes, are generated through different cellular machinery. Exosomes are derived from the endocytic pathway, where MVBs escape lysosomal degradation and fuse with the plasma membrane tor result in ILV secretion as exosomes. They accommodate biomolecules such as nucleic acids, proteins, lipids and metabolites. Ectosomes are generated by the outward budding of plasma membrane, while apoptotic bodies are formed by plasma membrane blebbing. Secretory autophagosomes are originated from the fusion of autophagosome-like vesicles with the plasma membrane. Migrasomes, unlike any other EVs, are formed on retraction fibers of migrating cells

The biogenesis of EVs, encompassing exosomes, ectosomes, apoptotic bodies, secretory autophagosomes/amphisomes, and migrasomes, reveals a sophisticated cellular machinery that governs their formation and cargo sorting (Fig. 1). Exosomes, originating from MVBs via the ESCRT-dependent or independent pathways (e.g., ceramide or tetraspanin-mediated), carry specific cargos influenced by their biogenesis mechanisms. For instance, the ESCRT-dependent pathway facilitates membrane remodeling and ubiquitinated cargo sorting, while the syndecan-syntenin-ALIX pathway offers an alternative route for exosome production. Similarly, ectosomes, formed through plasma membrane budding, share molecular machinery like ESCRT and tetraspanins, highlighting overlapping mechanisms with exosomes. Unique EV subtypes, such as migrasomes formed on retraction fibers of migrating cells, demonstrate distinct biogenesis pathways. These diverse and yet overlapping mechanisms emphasize the need for precise classification based on size, origin, and molecular content. These diverse biogenesis mechanisms determine the cargo landscapes of EVs, which in turn dictate their functional roles in intercellular communication, particularly within the TIME.

**Fig. 2:**
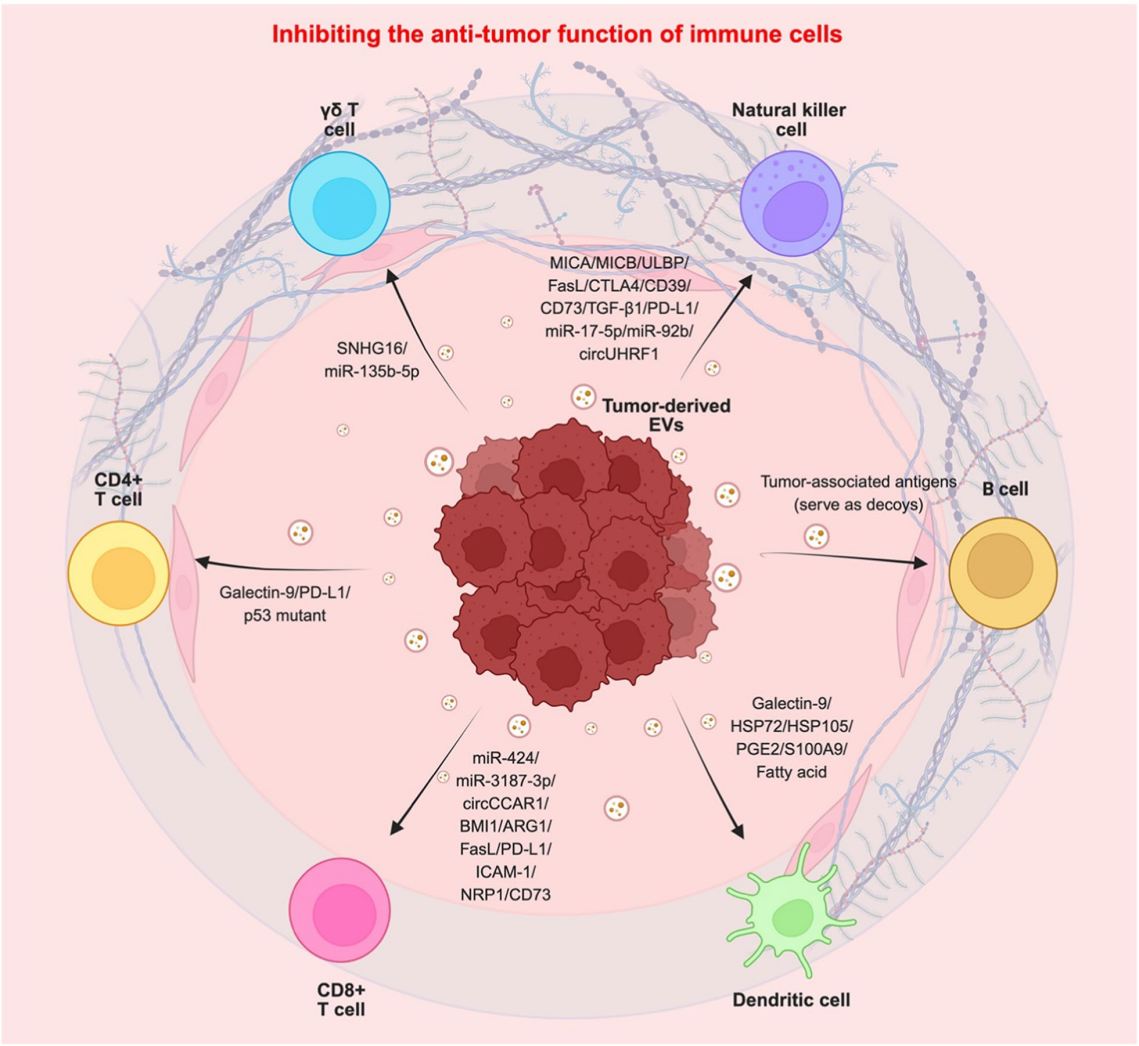
The effects of TEVs on the anti-tumor immune cells in TIME. TEVs inhibit anti-tumor immune cells to shape immunosuppressive TME. TEVs carry various cargos to suppress the functions of anti-tumor cells, such as CD8 + T cells, CD4 + T cells, γδ T cells, NK cells, B cells, and DCs to facilitate tumor progression

**Fig. 3:**
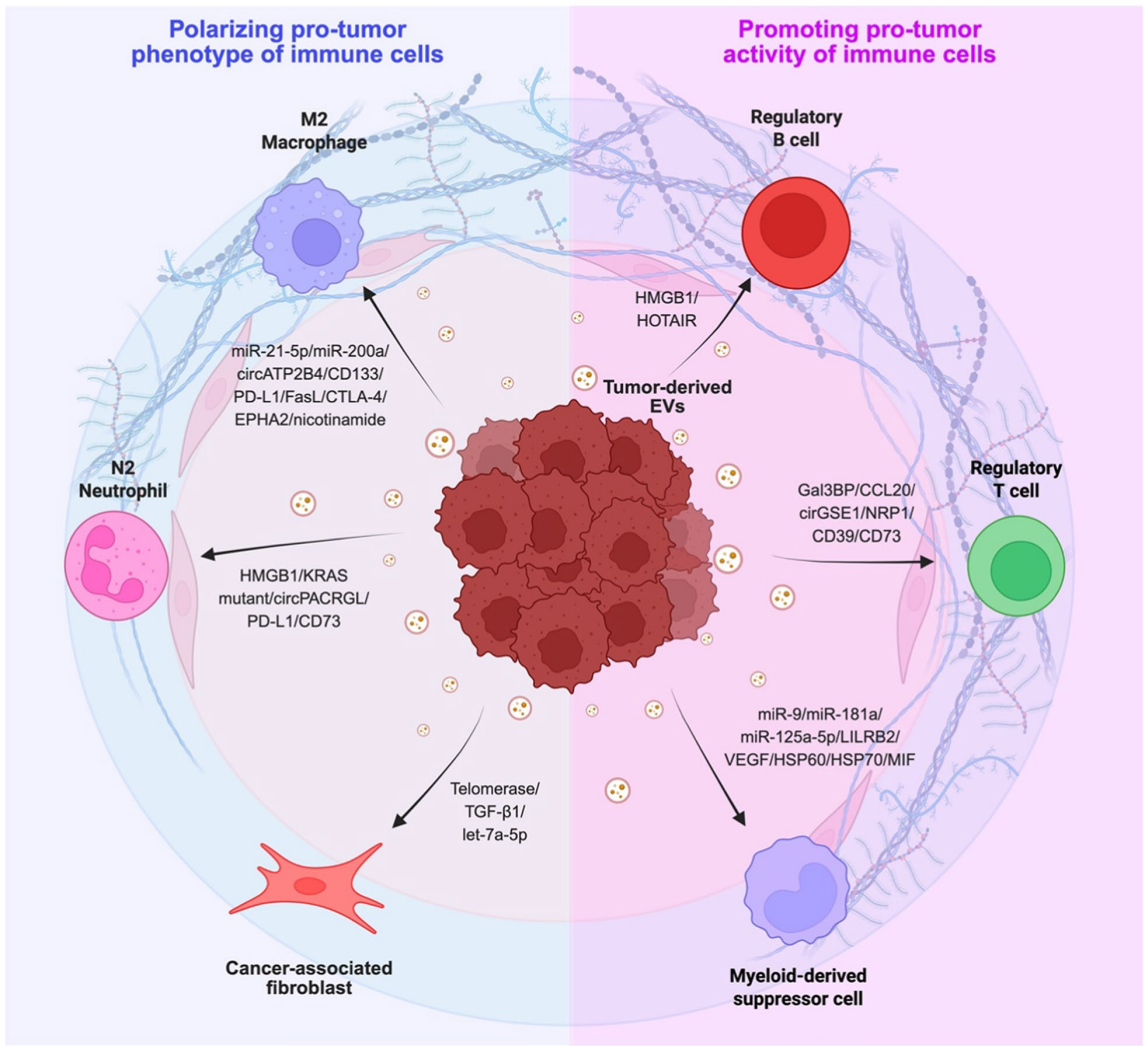
The effects of TEVs on the pro-tumor immune cells in TIME. TEVs promote the function of pro-tumor immune cells to shape immunosuppressive TME. TEVs carry various cargos to activate the pro-tumor cells, such as MDSCs, Tregs, and Bregs to mitigate the anti-tumor immunity. Moreover, by inducing pro-tumor polarization of macrophages, neutrophils, and CAFs, TEVs sustain the immunosuppressive TME and promote tumor immune evasion

**Fig. 4:**
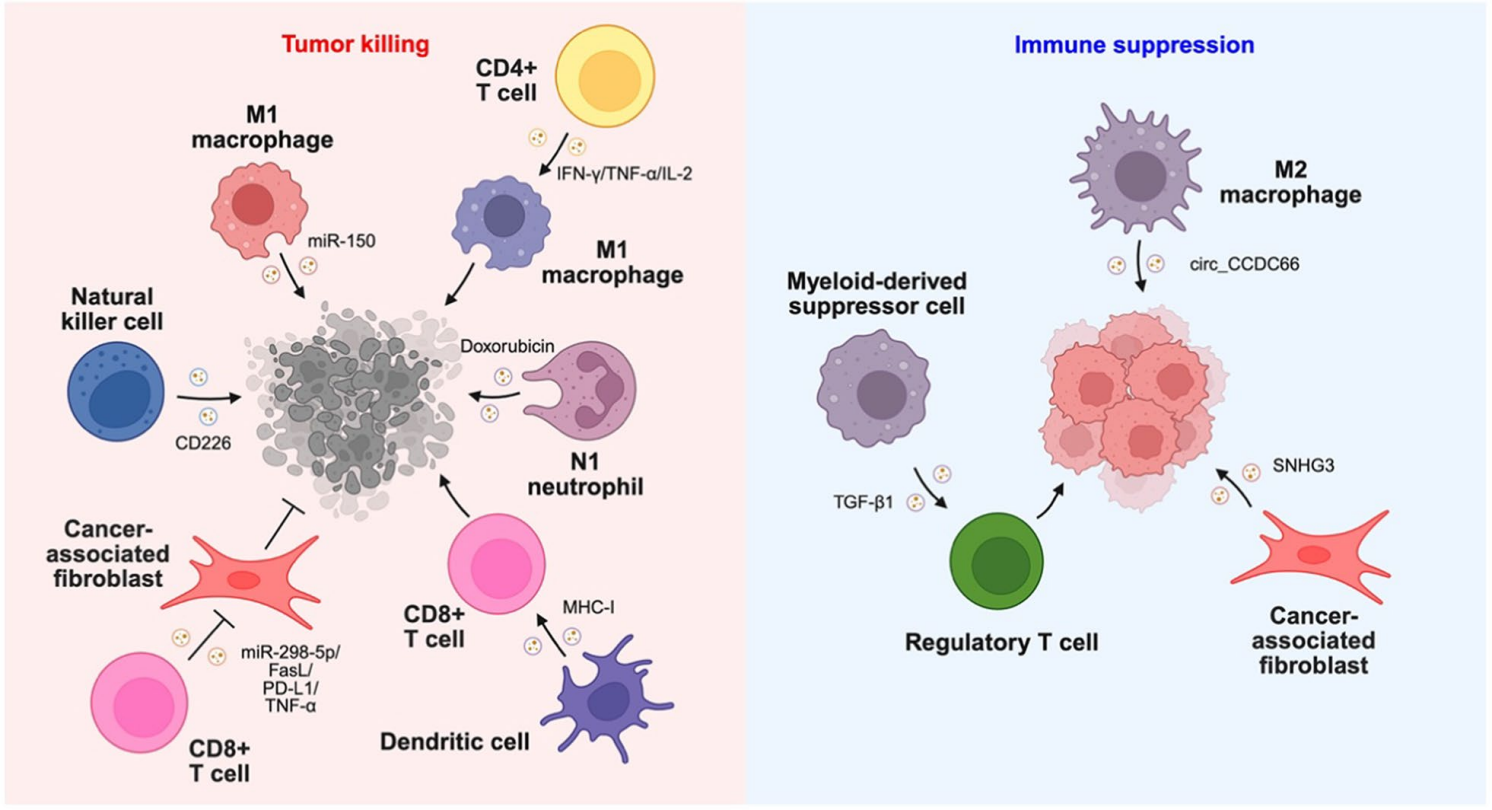
The effects of immune cell-derived EVs on TIME. Anti-tumor immune cells, such as CD4 + T cells, CD8 + T cells, M1 macrophages, N1 neutrophils, NK cells and DCs secrete EVs to reduce tumor progression, while pro-tumor immune cells such as M2 macrophages, MDSC and CAF secrete EVs to promote cancer growth through immune suppression

**Fig. 5:**
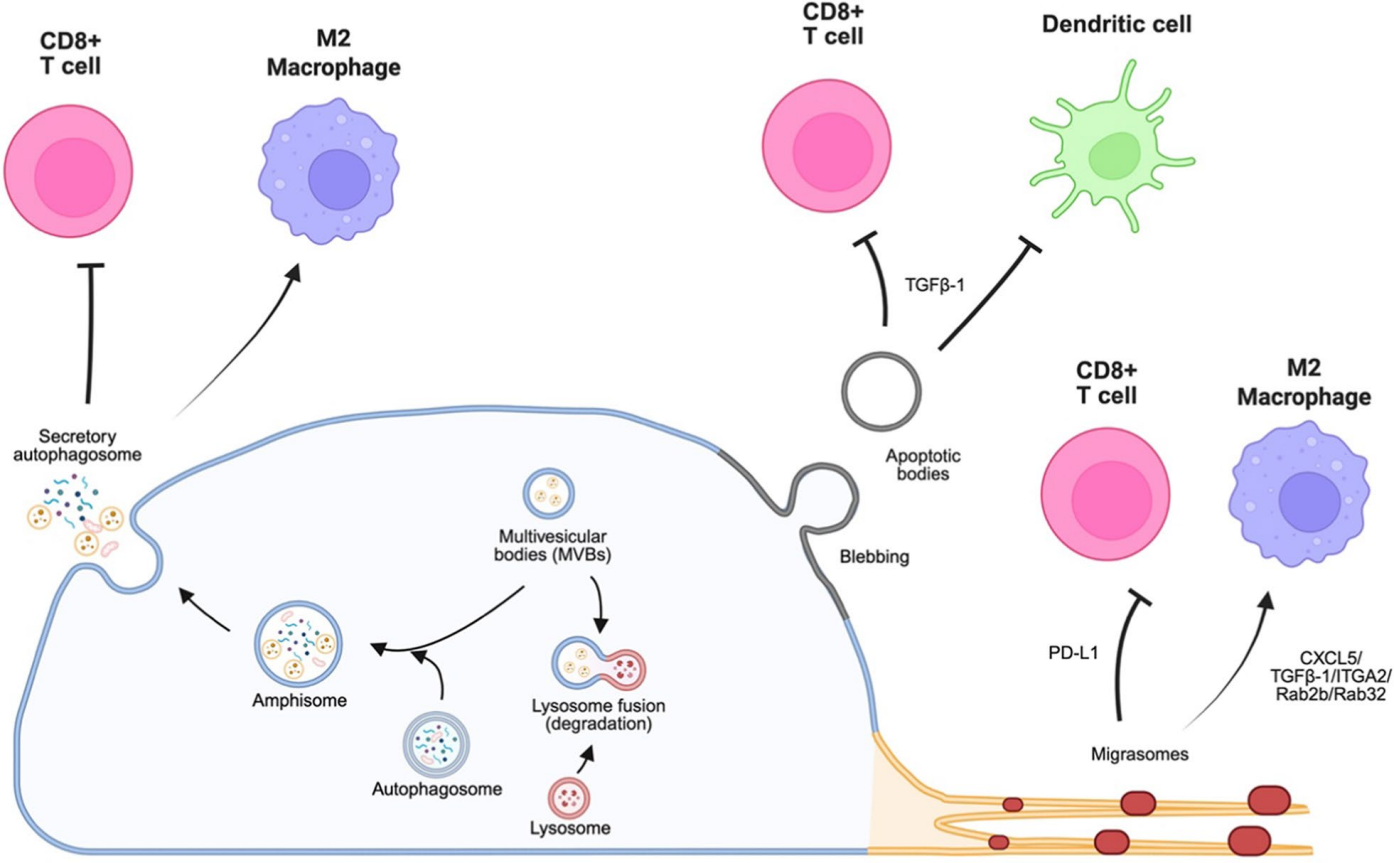
The effects of atypical EVs on TIME. Atypical EVs secreted by tumor cells, including apoptotic bodies, migrasomes and secretory autophagosomes suppress the anti-tumor function of CD8 + T cells and DCs, while promoting pro-tumor macrophage M2 polarization

In the TIME, TEVs orchestrate a pro-tumorigenic environment by suppressing the functions of anti-tumor cells, such as CD8 + T cells, CD4 + T cells, γδ T cells, NK cells, B cells, and DCs, and promoting the pro-tumor cells, such as MDSCs, Tregs, and Bregs, and inducing pro-tumor polarization of macrophages, neutrophils, and CAFs. TEVs are either taken up by immune cells or interact with immune cells through membrane-bound proteins to alter their functions. The cargos in tumor-derived EVs that impact on various anti-tumor and pro-tumor cells in TIMEs are summarized in Fig. 2 and Fig. 3, respectively. Reciprocally, anti-tumor immune cell-derived EVs, such as those from CD8 + T cells or M1 macrophages, exert anti-tumor effects by enhancing cytotoxic activity or inhibiting tumor stromal cell formation, while pro-tumor immune cell-derived EVs, such as those from MDSCs and M2 macrophages, promote tumor progression by dampening anti-tumor immunity. The impacts of various immune cell-derived EVs in TIME on tumor cells are illustrated in Fig. 4. These distinct outcomes highlight the bidirectional nature of EV-mediated communication in the TIME. Finally, the roles of several tumor-derived, atypical EVs in TIME regulation are summarized in Fig. 5.

The therapeutic potential of EVs is a burgeoning field with two primary strategies: inhibiting TEV biogenesis and cargo sorting to disrupt their immunosuppressive effects, or utilizing EVs as biocompatible drug delivery vehicles. Indeed, EVs’ natural biocompatibility makes them ideal for delivering therapeutic agents, such as curcumin, siRNAs, or tumor-associated antigens, as evidenced by ongoing clinical trials. These trials leverage plant-derived EVs, mesenchymal stromal cell-derived EVs, or DC-derived EVs to target various types of cancer, aiming to modulate oncogenic signaling or to enhance immune activation. The success of these trials could pave the way for EV-based therapies to become a cornerstone of precision oncology.

However, challenges remain in translating EV-based strategies into clinical practice. The heterogeneity of EVs complicates their isolation and characterization, necessitating standardized protocols to distinguish subtypes based on size, biogenesis, and cargo [265]. Moreover, the dual role of EVs in promoting or suppressing tumor growth, depending on their cellular origin and cargos, underscores the need for targeted interventions that selectively modulate TEVs without affecting beneficial immune cell-derived EVs. Future research should focus on elucidating the molecular determinants of EV fate (secretory vs. degradative) and developing scalable methods for EV engineering to enhance their therapeutic efficacy.

In conclusion, EVs are pivotal players in the TIME, orchestrating complex tumor-immune interactions that drive cancer progression or regression. Their biogenesis mechanisms, ranging from ESCRT-dependent to unique pathways like migrasome formation, highlight the diversity of EV subtypes and their tailored functions. TEVs reshape the TIME to favor immunosuppression, while immune cell-derived EVs offer potential anti-tumor effects. The therapeutic exploitation of EVs, whether by inhibiting their pro-tumorigenic roles or harnessing them as drug delivery platforms, holds immense promise for cancer immunotherapy. As research advances and clinical trials progress, EVs are poised to transform the landscape of cancer treatment, offering novel strategies to combat tumor immune evasion and to enhance patient outcomes.

## Data Availability

No datasets were generated or analysed during the current study.

## Abbreviations

AI: Artificial intelligence
APC: Antigen presenting cell
ARRDC-1: Arrestin domain-containing protein 1
ARRMs: ARRDC1-mediated microvesicles
Bregs: Regulatory B cells
CAF: Cancer-associated fibroblast
CT: Computed tomography
ccRCC: Clear cell renal cell carcinoma
circRNA: Circular RNA
CRC: Colorectal cancer
DC: Dendritic cell
ESCRT: Endosomal sorting complex required for transport
EV: Extracellular vesicle
Gal3BP: Galectin-3 binding protein
HCC: Hepatocellular carcinoma
I-BAR: Inverse BAR
ILV: Intraluminal vesicle
L_0_ domains: Liquid-ordered domains
LDELS: LC3-dependent extracellular vesicle loading and secretion
lncRNA: Long non-coding RNA
MDSC: Myeloid-derived suppressor cells
MFSs: Migrasome formation sites
miRNA: MicroRNA
MT: Microtubule
MVB: Multivesicular body
NK: Natural killer
NKG2D: Natural killer group 2D
NPC: Nasopharyngeal carcinoma
NSCLC: Non-small cell lung cancer
OS: Overall survival
PDAC: Pancreatic ductal adenocarcinoma
PFS: Progression-free survival
PIP5KIα: Phosphatidylinositol phosphate 5-kinase
piRNAs: Piwi-interacting RNA
PLP: Proteolipid protein
SERS: Surface-Enhanced Raman Scattering
TAM: Tumor-associated macrophage
TCR: T cell receptor
TEMs: Tetraspanin-enriched microdomains
TEMAs: Tetraspanin-enriched macrodomains
TEV: Tumor-derived extracellular vesicle
TIME: Tumor immune microenvironment
TLR: Toll-like receptor
TME: Tumor microenvironment
TNBC: Triple negative breast cancer
Treg: Regulatory T cell
Tspan4: Tetraspanin 4
